# Handheld Lab-on-a-Chip System for Label-Free Dual-Plex Detection of Biomarkers Through On-Chip Plasma Separation

**DOI:** 10.3390/bios15110743

**Published:** 2025-11-04

**Authors:** Chen-Yuan Chang, Yuan-Pei Lei, Chien Chieh Chiang, Cheng-Sheng Huang

**Affiliations:** Department of Mechanical Engineering, National Yang Ming Chiao Tung University, Hsinchu 30010, Taiwan; zyzhang.en11@nycu.edu.tw (C.-Y.C.); bernardlei.en11@nycu.edu.tw (Y.-P.L.); yt8h96k54@gmail.com (C.C.C.)

**Keywords:** lab-on-a-chip, label-free biosensor, guided-mode resonance, immunosensor

## Abstract

Rapid and reliable detection of biomarkers in complex fluids such as whole blood is essential for effective disease diagnosis and monitoring, particularly in point-of-care settings. Accordingly, this study developed a handheld lab-on-a-chip (LOC) platform that integrates on-chip plasma separation with label-free optical biosensing for real-time, dual-plex detection of biomarkers. The LOC platform includes a two-stage filtration unit that enables efficient separation of plasma from whole blood. This platform also includes a novel gradient grating period guided-mode resonance sensor array that is capable of simultaneously detecting multiple biomarkers with high sensitivity. A compact handheld reader was developed to acquire and analyze optical signals. By using creatinine and albumin as model biomarkers, we demonstrated that the developed platform could achieve sensitive, specific, and reproducible biomarker detection in both plasma and whole-blood samples. The platform can detect albumin and creatinine at concentrations as low as 0.8 and 1.44 μg/mL, respectively, and it exhibits minimal nonspecific binding. These results highlight the potential of the proposed system as a robust and accessible tool for decentralized diagnostics.

## 1. Introduction

The rising prevalence of chronic diseases and viral infections highlights the urgent need for a technology that enables rapid and accurate detection of biomolecules or biomarkers in complex biological fluids such as blood or urine. Timely diagnosis and effective disease monitoring are critical for clinical decision-making and for advancing point-of-care (POC) testing capabilities. Traditional diagnostic methods, such as enzyme-linked immunosorbent assays (ELISAs), often require labor-intensive sample preparation steps, including centrifugation and sample transportation. These processes can introduce variability, increase turnaround time, and hinder the accessibility of diagnostics, particularly in decentralized or resource-limited settings. To address these limitations, microfluidic technologies, which are commonly termed lab-on-a-chip (LOC) systems, have emerged as promising platforms for POC diagnostics and have attracted considerable research attention because of their potential advantages. These advantages include the ability to integrate multiple functional components or processes onto a single platform, fabricate batches at low cost, reduce sample volume requirements, enhance reaction kinetics in microscale channels, and automate fluid handling [1].

Biomarker concentrations in bodily fluids such as blood, urine, or saliva often reflect physiological changes or disease states. However, direct detection of these biomarkers is frequently hindered by blood cells or other interfering substances. Consequently, plasma separation or sample purification is typically required to improve assay sensitivity and reliability [2,3,4]. Various microfluidic strategies have been developed for on-chip blood cell separation, and these strategies are generally categorized into active and passive methods. Active methods rely on mechanisms such as centrifugal forces, dielectrophoresis, acoustic waves, or magnetic fields [5,6,7,8]. Although these methods are effective, they often require prior dilution of the blood sample. Moreover, the application of active forces may cause cell lysis, leading to the release of cellular debris into the plasma. Such debris can interfere with biomarker detection, resulting in false signals. In addition, the requirement of external power sources complicates the design and fabrication of LOC devices and limits their portability [9,10]. By contrast, passive separation methods do not require an external power source and rely on mechanisms such as pressure-driven flow, inertial forces, the Zweifach–Fung effect, sedimentation, and shear forces [11,12,13,14,15,16,17,18]. Passive separation methods have been successfully implemented in LOC systems for blood plasma separation. Despite their effectiveness, many of these methods exhibit certain drawbacks, including the need for complex microchannel designs or blood predilution [19]. Size-based membrane filtration is a simple and cost-effective passive method that can be easily integrated into on-chip systems for blood plasma separation. This method has been widely adopted in LOC platforms for various biomarker detection applications [19,20,21,22,23,24,25,26].

ELISA and fluorescence-based immunoassays remain the dominant techniques for serological biomarker detection and have been successfully incorporated into various LOC platforms for diverse applications [25,27,28,29]. However, label-free (LF) biosensing technologies offer several advantages over these techniques, including the simplification of sample preparation, reduction in assay times, and elimination of labeling steps that can introduce variability or affect biomolecular activity. Consequently, LF biosensors have attracted considerable attention and have become powerful tools for real-time, direct monitoring of biomolecular interactions. Among LF biosensors that employ various transduction methods, optical LF biosensors are particularly attractive because of their high sensitivity, electromagnetic interference resistance, compact design, simple optical readout, and multiplexing and remote-sensing capabilities [30]. Compared with electrical and electrochemical biosensors, optical LF biosensors offer improved reproducibility and exhibit pH-independent performance [25]. Various optical biosensors can quantify biomolecular concentrations through different modalities, including angular, intensity, wavelength, and phase modulation [31]. Sensors based on surface plasmon resonance and localized surface plasmon resonance are the most extensively studied optical LF biosensors because of their high sensitivity. However, these sensors typically have limited resolution owing to their broad full-width at half maximum (FWHM) at resonance, which results from substantial photon loss in the metal layer [32]. By contrast, dielectric guided-mode resonance (GMR) devices, which are also known as photonic crystals or resonant waveguide gratings, can achieve narrow bandwidths (<1 nm), enabling the detection of smaller resonant wavelength shifts and thus offering improved resolution. Therefore, dielectric GMR devices have been extensively investigated for biosensing applications [33,34].

Despite the complementary advantages of optical LF biosensors and microfluidic plasma separation, few studies have successfully integrated both functionalities into a single LOC platform capable of simultaneously isolating plasma and detecting biomarkers from whole-blood samples [22,25,35,36]. Accordingly, this study developed an LOC system that integrates a two-stage filtration unit with an optical LF biosensor for the simultaneous detection of albumin and creatinine directly from whole blood. A portable, handheld optical reader was used for signal acquisition, demonstrating the feasibility of this integrated system for real-time, multiplexed biomarker detection.

## 2. Materials and Methods

**Figure 1:**
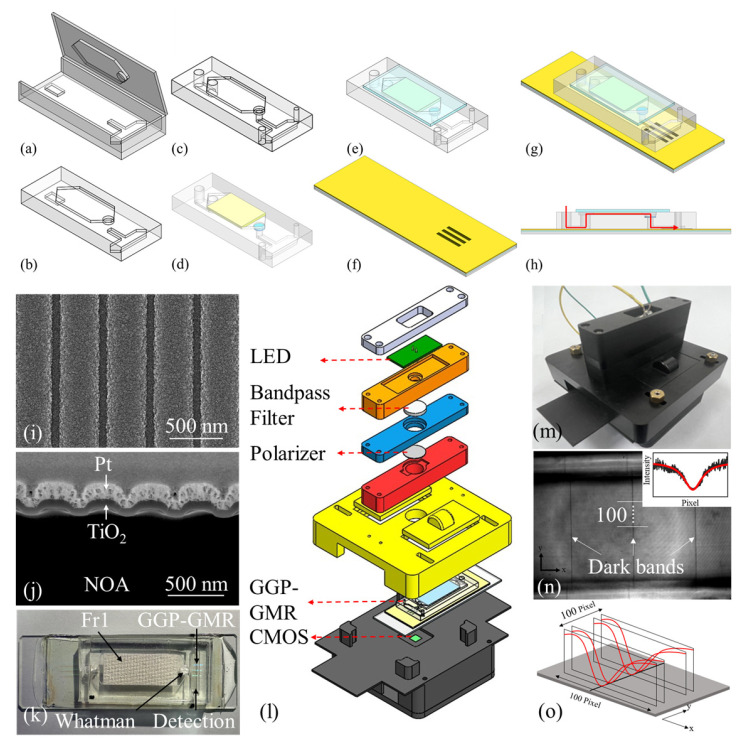
(**a**–**h**) Schematic of the overall fabrication process for the developed LOC device. (**i**) Top and (**j**) cross-sectional views of the fabricated GGP-GMR sensor array. (**k**) Photograph of the complete LOC device. (**l**) Exploded-view schematic of the adopted handheld device. (**m**) Photograph of the assembled handheld device. (**n**) Complementary metal–oxide–semiconductor (CMOS) image showing three distinct dark bands corresponding to three GGP-GMR sensors in the array; inset: intensity distribution along one of the white transverse lines. (**o**) Final dark band position determined by averaging 100 transverse intensity profiles.

The optofluidic chip, which constitutes the core component of the proposed LOC system, was fabricated through a four-step process: microfluidic channel fabrication [Figure 1a–c], membrane integration [Figure 1d,e], gradient grating period (GGP)-GMR sensor array fabrication [Figure 1f], and final microfluidic channel–GGP-GMR sensor array bonding [Figure 1g,h].

### 2.1. Design and Fabrication of Microfluidic Chip

Inspired by previous studies [20,21,26], this study developed a two-stage filtration unit to enable efficient blood plasma separation. First, a blood filtration membrane with relatively large pore sizes (FR1, Advanced Microdevices Ltd., Ambala Cantt, India) was used to facilitate the initial separation of blood cells [26]. Subsequently, a second filtration membrane (Whatman filter paper, Grade GF/A, Merck KGAA, Darmstadt, Germany) with smaller pore sizes (~1.6 μm) was employed to remove residual debris and cell pellets. To fabricate the microfluidic channel, polymethyl methacrylate (PMMA) was used to construct top and bottom molds [Figure 1a] through computer numerical control (CNC) micromilling. Polydimethylsiloxane (PDMS; SYLGARD 184 Silicone Elastomer Kit, Dow Corning, Midland, MI, USA) was then prepared by thoroughly mixing the base and curing agent in a 10:1 ratio. After the completion of the degassing process, liquid PDMS was gently poured into the assembled PMMA molds. The PDMS–PMMA structures were then cured in an oven at 80 °C for 1 h, after which they were carefully demolded [Figure 1b]. Vertical channels connecting the top and bottom layers as well as the inlet and outlet ports were created using biopsy punches of various diameters. The FR1 and Whatman membranes were then placed at their designated locations within the microfluidic channels [indicated by yellow and blue, respectively, in Figure 1d]. Finally, the top layer was sealed using a thin layer (thickness of ~1 mm) of PDMS sheet [Figure 1e].

### 2.2. Design and Fabrication of GGP-GMR Sensors

An appropriately designed GMR sensor resonates when light of a specific wavelength is incident on it, with maximum reflection and minimal transmission occurring under normal incidence [37,38]. The resonant wavelength can be determined using the second-order Bragg condition [39], which is expressed as follows:

(1) $$\lambda_{R}=n_{eff}\Lambda$$

where $\lambda_{R}$ is the resonant wavelength, $n_{eff}$ is the effective refractive index (RI), and Λ is the grating period. The effective RI ($n_{eff}$) can be considered a weighted average of the RIs of the overall GMR structure, including its substrate, waveguiding, and cladding layers. This parameter can be calculated using the method described by [40]. Any change in sample concentration (or RI) within the cladding layer causes a change in $n_{eff}$, which results in a shift in the resonant wavelength ($\lambda_{R}$).

In contrast to conventional GMR sensors, which have a fixed grating period, the GGP-GMR sensors fabricated in this study exhibit a gradient grating structure, with grating periods ranging from 430 to 442 nm in 2 nm increments. Each period is repeated 100 times, creating a spatially varying grating along the device. When illuminated by a narrowband light source, the fabricated GGP-GMR sensors exhibit resonance only if the grating period satisfies the Bragg condition [Equation (1)]. Light is reflected through an area where this condition is satisfied, whereas it is transmitted through the other areas where the condition is not satisfied. A CMOS beneath GGP-GMR sensor detects a spatially varying intensity pattern, and the pixel corresponding to the area with the resonant grating period exhibits the lowest intensity. Variations in the properties of samples placed on a GGP-GMR sensor, such as variations in biomolecule concentration, cause changes in the effective RI of the sensor, which leads to a shift in its resonant grating period. A shift in the resonance grating period results in a change in the pixel corresponding to the lowest transmission intensity, which can be correlated with changes in the sample’s RI. Additional details on the design and properties of GGP-GMR sensors are provided in our previous work [41]. In the present study, a sensor array consisting of three GGP-GMR sensors was fabricated to achieve dual-plex detection capability. First, electron beam lithography and dry etching were employed to generate a suitable gradient grating pattern on a Si wafer. The prepared pattern was then transferred onto an optical adhesive [Norland Optical Adhesive (NOA) 13825; Norland Products, Jamesburg, NJ, USA] through replica molding by using polyethylene terephthalate (PET) as a flexible substrate. Subsequently, the NOA/PET film was bonded to a glass slide by using ultraviolet (UV)-curable adhesive. Finally, a thin layer of TiO_2_ was deposited onto the NOA surface through sputtering to complete the sensor structure [Figure 1f]. Figure 1i,j show the top and cross-sectional views, respectively, of the fabricated GGP-GMR sensor, which has a representative grating period of 436 nm, a grating depth of 80 nm, and a TiO_2_ layer thickness of 88 nm. Note that the Pt layer on top of the TiO_2_ is only used for SEM imaging to reduce charging effects and enhance image quality. The detailed fabrication procedure is described in our previous study [42].

### 2.3. Integration of LOC Device and Detection Setup

Uncured PDMS was used as an adhesive to bond the fabricated microfluidic channel to the fabricated GGP-GMR sensor array. Liquid PDMS with a base-to-curing agent ratio of 10:3 was thoroughly mixed, degassed, and spin-coated onto a glass substrate at 3000 rpm for 60 s. The fabricated microfluidic chip [Figure 1e] was gently pressed onto the spin-coated PDMS layer to add a thin film of PDMS to this chip. The chip was then carefully placed on the GGP-GMR sensor array. The assembled LOC device was subsequently placed in the oven at 70 °C for 60 min to complete the bonding process [Figure 1g,k]. Figure 1h illustrates a schematic side view of the assembled LOC device, where the red line represents the blood flow.

A portable handheld device was developed to read the optical response of the GGP-GMR sensor array. This device was fabricated using a method similar to that reported in our previous work [42]. Briefly, a light-emitting diode (KED35 RHD, Kyoto Semiconductor, Kyoto, Japan) light source combined with a narrow band-pass filter (Alluxa, Santa Rosa, CA, USA) was used to illuminate the handheld device. The resulting illumination has a center wavelength of 656.8 nm and an FWHM of 1 nm. To achieve high detection sensitivity, the handheld device incorporated transverse magnetic polarization control. A complementary metal–oxide–semiconductor (CMOS) imaging sensor (SME-B050-U, MIGHTEX, Pleasanton, CA, USA) with a resolution of 2560 × 1920 pixels and a pixel size of 2.2 × 2.2 µm^2^ was used to capture the light transmitted through the GGP-GMR sensor array. Figure 1l presents an exploded-view schematic of the handheld device, and Figure 1m depicts a photograph of the final assembled handheld device, which was manufactured through CNC machining.

When the narrowband light source illuminates the GGP-GMR sensor array, the light resonates at a location with a specific period, resulting in it being reflected at that location. This phenomenon results in a localized drop in transmission intensity, which appears as dark bands in the CMOS camera. Because the GGP-GMR sensor array developed in this study contains three sensors [Figure 1f,k], three distinct dark bands are captured by the CMOS camera [Figure 1n]. The inset of Figure 1n displays the intensity profile along one of the white transverse lines in this figure. To accurately identify the position of each dark band, the raw data were smoothed and fitted to a Gaussian model by using MATLAB’s smoothdata function (R2019b, MathWorks, Natick, MA, USA). The pixel corresponding to the minimum intensity in the fitted curve represents the center of the dark band. This approach enables subpixel resolution in locating resonance shifts. To reduce experimental uncertainty, the final dark band position was calculated as the average of 100 transverse intensity profiles, as illustrated in Figure 1o.

### 2.4. Chip Preparation for Detection of Multiple Biomarkers

Testing blood for various substances, such as proteins, nucleic acids, and metabolites, is an essential practice in modern medicine that enables disease diagnosis, disease monitoring, and personalized treatment. For example, serum creatinine concentration is commonly used to determine the stage of chronic kidney disease [43,44], and abnormal serum albumin concentrations may indicate cancer, liver failure, or chronic hepatitis [45,46]. In the present study, albumin and creatinine were selected as model biomarkers to demonstrate the feasibility of the proposed detection system. An immunoassay approach was employed for biomarker recognition. Before the microfluidic channel was bonded with the GGP-GMR sensor array, the sensor surface was treated with oxygen plasma to enrich it with hydroxyl groups [47], thereby enhancing the efficiency of surface functionalization. A 2% solution of epoxy silane (3-glycidoxypropyl dimethoxysilane in toluene) was then applied on the sensor surface to form covalent bonds with hydroxyl groups through silanization [48].

Antialbumin antibodies (ab10241, Abcam, Cambridge, UK) and anticreatinine antibodies (ab30719, Abcam) were diluted in phosphate-buffered saline (PBS) to a concentration of 100 µg/mL. The antialbumin and anticreatinine antibody solutions were then carefully injected into the GGP-GMR region from the left and right inlet ports, respectively, such that antialbumin and anticreatinine antibodies were only immobilized on the two outer GGP-GMR sensors, with the central sensor remaining unmodified to serve as a reference. The LOC device was then incubated at 4 °C for 12 h to enable antibody immobilization. Subsequently, the antibody solutions were aspirated, and the microfluidic channel was thoroughly rinsed with PBS-T (0.05% Tween 20 in PBS) and then PBS to remove any unbound antibodies. To minimize nonspecific binding in subsequent steps, the sensors were blocked with 1% casein in PBS (Casein Blocker, Bio-Rad, Hercules, CA, USA) for 1 h. After blocking was completed, the solution was removed, and the channel was rinsed again with PBS-T and then PBS. Next, fresh PBS was injected into the channel, and the sensor responses were recorded with the handheld device, with images captured every 10 s for 5 min. The positions of the dark bands in each image were determined using the methods described in the previous section, and the average position across all images was used as the baseline signal for the three GGP-GMR sensors.

## 3. Results

### 3.1. Filtration by a Two-Stage Unit

**Figure 2:**
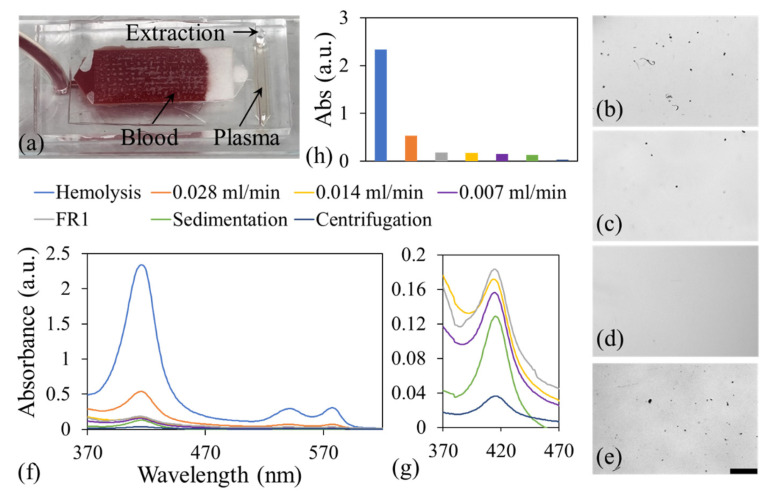
(**a**) Photograph showing blood flow through the developed LOC device. Microscopic images of the plasma region when blood was passed through both the FR1 and Whatman membranes at flow rates of (**b**) 0.028, (**c**) 0.014, and (**d**) 0.007 mL/min. (**e**) Microscopic image of the plasma region when blood was passed through only the FR1 membrane at a flow rate of 0.007 mL/min. (**f**) Absorbance spectra of plasma samples obtained using different separation methods. (**g**) Enlarged view of the absorbance spectra in (**f**) at 370–470 nm. (**h**) Absorbance of different samples at 415 nm. Scale bar in (**e**) applied to (**b**–**e**). The color legends in (**f**) also apply to (**g**) and (**h**).

In this study, rabbit blood (purchased from GeneTex, Taiwan), whose red blood cells are similar in size to those of humans, was used to evaluate the filtration efficiency of the proposed system and to simulate biomarker detection for human samples. Whole blood was introduced into the microfluidic channel of the proposed system by using a syringe pump (Fusion 100, Chemyx Inc., Stafford, TX, USA) at various flow rates to determine the optimal operational conditions [Figure 2a]. After the blood passed through the FR1 and Whatman filter membranes, the plasma region (or detection region) was examined using an inverted microscope (IX73, Olympus) to assess hemolysis and debris levels. Figure 2b–d) depict microscopic images of the plasma region observed at injection flow rates of 0.028, 0.014, and 0.007 mL/min, respectively. At the lowest flow rate of 0.007 mL/min, minimum cellular debris was observed, indicating lower hemolysis compared with that at higher flow rates. In a control experiment conducted at the flow rate of 0.007 mL/min without the Whatman membrane, a substantial increase in debris was observed [Figure 2e], which confirmed the critical role of the secondary filtration membrane in improving plasma purity.

Hemoglobin exhibits characteristic absorption peaks at 415, 541, and 576 nm [49]. Therefore, in addition to microscopic observation, a UV–visible spectrophotometer (A688, Multiskan) was used to analyze plasma samples to assess the degree of hemolysis. The absorbance spectra of the filtered plasma samples are illustrated in Figure 2f. A hemolyzed control sample, which was prepared through vigorous hand-shaking of whole blood, exhibited distinct absorption peaks at 415, 541, and 576 nm, consistent with previously reported data [49]. For comparison, plasma samples obtained through two conventional separation methods, namely centrifugation (10,000× *g* for 10 min) and sedimentation (4 °C for 24 h), were also assessed; both samples showed lower absorbance levels than did the filtered samples. Figure 2g depicts a magnified view of the absorbance spectra of different plasma samples at 370–470 nm, and Figure 2h indicates the absorbance at 415 nm for different samples. At 415 nm, the plasma sample injected at a flow rate of 0.028 m/min exhibited high absorbance, indicating high hemoglobin content resulting from red blood cell lysis, which was likely caused by excessive shear stress at the flow rate of 0.028 mL/min. The samples processed at lower flow rates showed lower absorbance. Notably, at a flow rate of 0.007 mL/min, the plasma filtered through both the FR1 and Whatman membranes exhibited substantially lower absorbance than did that filtered through the FR1 membrane alone, confirming the effectiveness of the two-stage filtration system in minimizing hemolysis and improving plasma purity. The absorbance measurements were consistent with the microscopic observations, with higher levels of visible debris correlated with higher absorbance. In summary, the use of both filter membranes at an injection flow rate of 0.007 mL/min resulted in minimal hemolysis. Therefore, this condition was selected for subsequent biomolecule detection experiments. It should be noted that even with two-stage filtration at a flow rate of 0.007 mL/min, the absorbance remains higher than that obtained from sedimentation and centrifugation, indicating minor hemolysis. In addition, although the average diameter of platelets is between 2 and 4 μm, some platelets can be smaller than 1.6 μm. Both factors may introduce noise in detection and reduce detection sensitivity. This issue could be further investigated by employing filtration membranes with smaller pore sizes or reducing the injection flow rate. In the current demonstration, a syringe pump was used to characterize the required sample flow rate that minimizes hemolysis. However, using a syringe pump for sample delivery may not be practical for POC applications. Therefore, further integration of a flow-driving module or sample delivery mechanism into a microfluidic system without the need for bulky sample delivery equipment is necessary to make it more suitable for practical POC use.

### 3.2. Sucrose Measurement

**Figure 3:**
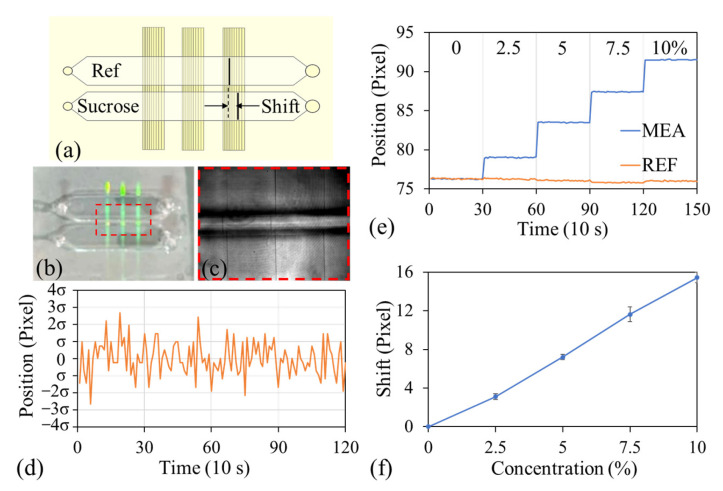
(**a**) Schematic and (**b**) top-view photograph of a two-channel microfluidic chip in which each channel is embedded with three GGP-GMR sensors. (**c**) Six dark bands observed across the two channels. (**d**) Fluctuation of the dark band location of one sensor around the mean location. (**e**) Dark band positions at various sucrose concentrations. (**f**) Average net shifts in dark band position at different sucrose concentrations.

This study designed a simple two-channel microfluidic chip, with each channel embedded with three GGP-GMR sensors [Figure 3a,b]. This chip was used to evaluate the sensing performance of the GGP-GMR sensors with signal readout by the handheld device. When narrowband light illuminated the GGP-GMR sensors, dark bands were observed by the underlying CMOS [Figure 3c]. To assess signal stability, deionized (DI) water was injected into one of the channels, and 120 images were captured at 10 s intervals over 20 min. The dark band location was determined for one of the sensors by using the method described previously. The fluctuation of the dark band location centered about the mean location is shown in Figure 3d. The position measurement noise, which was defined as three times the standard deviation, was determined to be 0.124 pixels (or 0.273 μm). The resolution reflects the overall detection system that can be achieved. It is determined by a combination of factors, including intrinsic resonant characteristics of GGP-GMR sensor, the optical design of the portable device, and the intrinsic electronic noise of the CMOS sensor. At this stage, we believe that further optimization of the GGP-GMR sensor can enhance the detection resolution. Two strategies can be explored: (1) optimizing the GMR structure to achieve a narrower resonance, which would narrow the dark band observed in the CMOS and improve detection resolution; and (2) increasing the sensitivity of the GGP-GMR sensor so that a change in reflective index produces a larger shift in the resonant wavelength.

Sucrose solutions with various concentrations were used to evaluate the bulk sensitivity and limit of detection (LOD) of the developed LOC device, which were measured using the handheld device. Initially, both channels were filled with DI water, and the resulting signals were used as a baseline. The DI water was then aspirated, and sucrose solutions at concentrations of 2.5%, 5%, 7.5%, and 10% were sequentially injected into one of the channels (measurement channel, MEA). This channel was rinsed with DI water between injection steps. The other channel served as a reference (REF), containing only DI water throughout the experiment. Figure 3e illustrates the dark band position at each sucrose concentration. The net shift in the dark band at each sucrose concentration relative to the dark band at 0% sucrose concentration (or DI water) was obtained by subtracting the dark band positions in the two channels, with three datasets acquired per chip. The average net shift at each sucrose concentration is displayed in Figure 3f. Increasing the sucrose concentration from 0% to 10% resulted in an average net shift of 15.43 pixels, which was equivalent to 33.95 μm. This increase in sucrose concentration also caused a 0.01454-RIU change in the RI, which was measured using a commercial refractometer (J47-HA, Rudolph Research Analytical, Hackettstown, NJ, USA). The average sensitivity of the LOC device was calculated to be 2334.86 μm/RIU by dividing the shift in the dark band position by the change in RI. Moreover, the LOD of the device was 1.17 × 10^−4^ RIU, which was determined by dividing the measurement noise by the sensitivity.

### 3.3. Whole-Blood and Plasma Measurements

In this study, the levels of albumin and creatinine spiked into rabbit whole-blood and plasma samples were measured to validate the effectiveness of the proposed LF biomarker detection approach with a two-stage filtration process. The developed LOC device contains three GGP-GMR sensors. The two outer sensors were immobilized with antibodies specific to albumin and creatinine for measurement (MEA), whereas the central sensor without antibodies served as a reference (REF). Creatinine (ab143309, Abcam) and albumin (ab205808, Abcam) were spiked into whole-blood and plasma samples such that the creatinine and albumin concentrations of these samples were 900 and 500 μg/mL, respectively. Plasma was obtained by centrifuging whole blood at 10,000 × *g* for 10 min by using a centrifuge (Centrifuge 3740, Kubota), after which the supernatant was extracted. Whole-blood and plasma samples with four additional creatinine and albumin concentrations were prepared through serial fivefold dilution.

**Figure 4:**
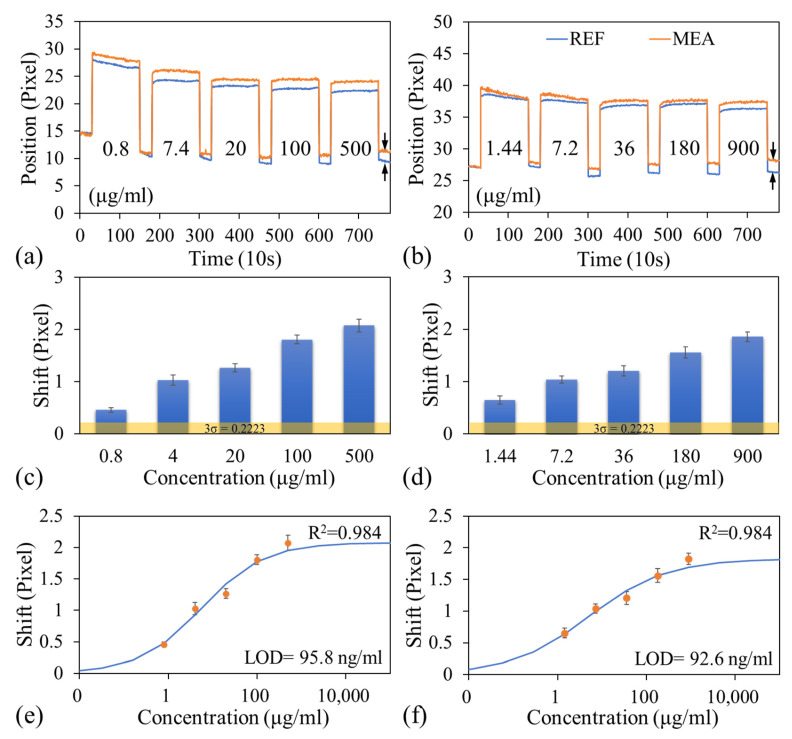
Variations in dark band positions over analyte concentration for the reference and measurement sensors in the measurement of (**a**) albumin and (**b**) creatinine levels. Variations in the average net shift in dark band position with the (**c**) albumin and (**d**) creatinine concentrations. Dose–response curves for (**e**) albumin and (**f**) creatinine in plasma.

To measure the albumin and creatinine levels in the plasma samples, PBS was first injected into the microfluidic channel, and images were captured every 10 s for 5 min to establish baseline dark band locations. Next, the PBS was aspirated, and a plasma sample containing the lowest concentration of albumin and creatinine was injected into the microfluidic channel. Images were then captured every 10 s for 20 min. Subsequently, the plasma sample was aspirated, and the channel was rinsed with PBS-T and then PBS. Finally, fresh PBS was injected into the channel, and images were captured every 10 s for 5 min to complete the concentration measurement. This procedure was conducted for each pair of albumin and creatinine concentrations. Figure 4a,b display the variations in dark band positions of MEA and REF for different analyte concentrations in representative experimental runs for albumin and creatinine, respectively. The arrows indicate the relative dark band shift between the MEA and REF sensors after rinsing the plasma and refilling the channel with fresh PBS. As the analyte concentration increases, more antigens bind to the antibodies on the sensor surface, resulting in a greater change in the surface refractive index and, consequently, a larger shift in the dark band location.

The net shift in the dark band, which resulted from analyte binding to the immobilized antibodies, was calculated by subtracting the shift observed in the reference sensor from that of the measurement sensor. This correction accounts for nonspecific binding and minor fluctuations caused by environmental or procedural variations. The experiment was repeated in two additional runs by using two separate LOC devices. Figure 4c,d presents the variations in the average net shift in the dark band position with the albumin and creatinine concentrations, respectively. These figure parts also illustrate the standard deviations of net shift over three independent runs, with the noise level (0.22 pixels) displayed for reference. The noise level was determined using the same approach as that used for the sucrose solution [Figure 3d].

A four-parameter logistic model was used to fit these experimental data using OriginPro 2016, and the fitted curves are shown in Figure 4e,f. along with the experimental data. The LOD for albumin and creatinine in plasma was determined as the concentration corresponding to the dark band shift associated with the blank concentration plus three times the average standard deviation of the dark band shifts obtained from all measured concentrations across all experimental runs. The calculated LODs for albumin and creatinine were 95.8 and 92.6 ng/mL, respectively.

The experimental procedure for whole-blood measurements was identical to that for plasma measurements, except that the blood was passed through a two-stage filtration unit to remove blood cells, allowing plasma containing target proteins to reach the GGP-GMR sensors for detection. In addition, each LOC device was used only once per sample measurement.

**Figure 5:**
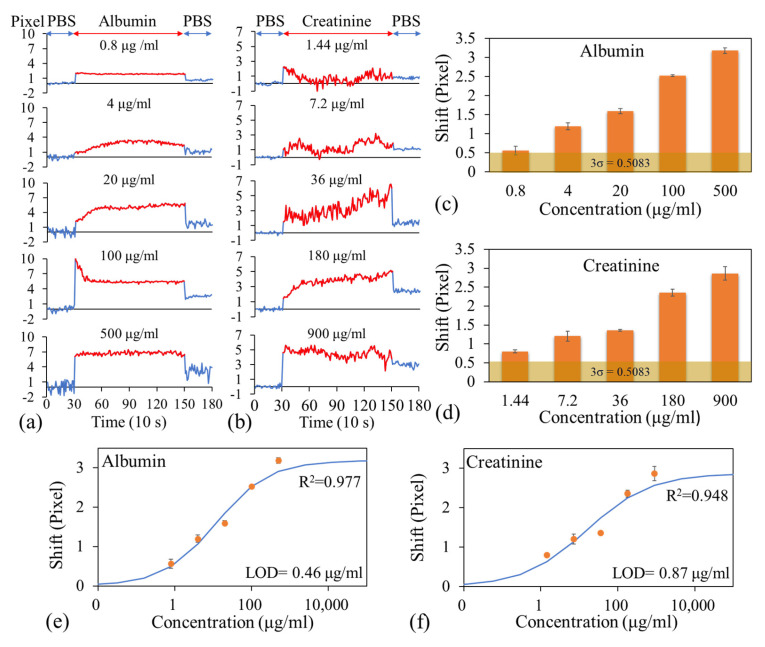
Variations in the net shift in the dark band position with time for different (**a**) albumin and (**b**) creatinine concentrations. Average net shifts in the dark band positions at different (**c**) albumin and (**d**) creatinine concentrations. Dose–response curves for (**e**) albumin and (**f**) creatinine in blood.

Figure 5a,b illustrate the variations in the net shift in the dark band position with time for different albumin and creatinine concentrations, respectively. Figure 5c,d present the average net shifts in the dark band positions at different albumin and creatinine concentrations, respectively, with the noise level included for reference. Notably, the net shifts observed at the lowest tested albumin and creatinine concentrations exceeded the noise threshold, indicating that the proposed detection system (comprising the developed LOC device and a handheld reader) is capable of detecting albumin and creatinine at concentrations as low as 0.8 and 1.44 μg/mL, respectively. Note that optimizing assay protocols and antibody binding strategies can further improve detection sensitivity, resolution, and dynamic range. The dose–response curves obtained from the four-parameter logistic model fitting are shown in Figure 5e,f. The LODs for albumin and creatinine were 0.46 and 0.87 μg/mL, respectively, which are much higher than those obtained from plasma samples. This is expected based on the discussion in Section 3.1, as the two-stage filtration is not completely free from hemolysis, and some platelets may pass through into the detection zone, thereby compromising detection sensitivity.

### 3.4. Selectivity Verification

**Figure 6:**
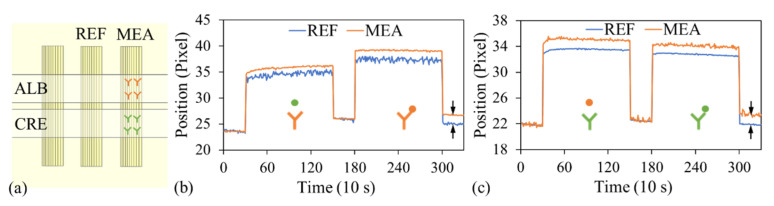
(**a**) Schematic of the LOC device used for nonspecific binding tests. Variations in dark band positions with time for the measurement (MEA) and reference (REF) sensors in the (**b**) albumin (ALB) and (**c**) creatinine (CRE) channels. Y-shaped symbols represent antibodies immobilized on the sensor surface, while circular dots indicate biomarkers present in the sample. Orange color denotes albumin or albumin-specific antibodies, and green color denotes creatinine or creatinine-specific antibodies.

A nonspecific binding test was conducted on a two-channel optofluidic chip [Figure 6a]. Within each channel, one outer GGP-GMR sensor was immobilized with antialbumin antibodies (MEA sensor in the albumin channel) or anticreatinine antibodies (MEA sensor in the creatinine channel), with the middle sensor serving as a reference (REF). Fresh PBS was first introduced into albumin channel to establish the baseline signal. Subsequently, 900 μg/mL of creatinine, which was spiked into centrifuged plasma, was injected into the albumin channel and incubated for 20 min. This channel was then sequentially rinsed with PBS-T and PBS, after which fresh PBS was injected into the channel, and images were captured every 10 s over a 5 min period. Next, 500 μg/mL of albumin, which was also spiked into centrifuged plasma, was injected into the albumin channel and incubated for 20 min. Finally, the sample was aspirated, the channel was rinsed with PBS-T and PBS, and images were captured every 10 s over a 5 min period after the channel was filled with fresh PBS. Figure 6b depicts the variations in the dark band locations with time for the MEA and REF sensors in the albumin channel. No notable relative shift in dark band position was observed between these sensors during creatinine exposure, indicating negligible nonspecific binding between creatinine and antialbumin antibodies. By contrast, a clear relative shift in dark band position was observed following albumin injection, as highlighted by the arrows in Figure 6b, confirming specific binding between albumin and its antibodies.

A similar experiment was performed in the creatinine channel. After baseline signals were established for the MEA and REF sensors, 500 μg/mL of albumin in plasma was injected into the creatinine channel and incubated for 20 min, aspirated and thoroughly rinsed with PBS-T and PBS, after which PBS was introduced into it. As displayed in Figure 6c, no substantial relative shift in dark band position was observed between the MEA and REF sensors, indicating minimal nonspecific binding between albumin and anticreatinine antibodies. Subsequently, the PBS was removed and 900 μg/mL of creatinine in plasma was injected into this channel and incubated for 20 min. After the final rinsing of the creatinine channel, PBS was injected into the channel, and images were recorded. A clear relative shift in dark band position was observed between the MEA and REF sensors [Figure 6c]. This shift confirmed that specific binding occurred between creatinine and its antibodies. It should be noted that for more practical and clinically relevant applications, cross-reactivity tests with other abundant plasma proteins should be conducted to evaluate the assay’s specificity and ensure that nonspecific binding does not interfere with the detection accuracy.

## 4. Conclusions

This study developed an LOC platform that integrates an efficient on-chip blood plasma separation system with an LF optical biosensor for dual-plex biomarker detection; we also validated its performance. The two-stage, membrane-based filtration unit incorporated into this platform effectively minimizes hemolysis, thereby enabling direct biomarker detection from whole blood without preprocessing. The LOC platform contains a GGP-GMR sensor array, which is paired with a handheld optical reader. The UV-curable polymer replica molding was employed to fabricate the GGP-GMR sensors. This approach enables batch production and makes the overall LOC device cost-effective for disposable use. Experimental results reveal that this system exhibited high sensitivity and specificity in detecting albumin and creatinine, which are clinically relevant biomarkers. The device also achieved subpixel resolution in resonance shift detection and had an LOD value of 1.17 × 10^−4^ RIU. It accurately measured albumin and creatinine concentrations as low as 0.8 and 1.44 μg/mL, respectively. Furthermore, nonspecific binding tests confirmed the selectivity of the immunoassay configuration of the proposed system. Overall, the simplicity, portability, and reliability of the proposed system make it suitable for POC applications, particularly in resource-limited environments. Our future research will focus on expanding the biomarker panel, enhancing system automation, and reducing system size to facilitate convenient field deployment of the proposed system.

## Data Availability

Data available on reasonable request from the corresponding author.
